# Socioeconomic inequalities in metabolic syndrome and its components in a sample of Iranian Kurdish adults

**DOI:** 10.4178/epih.e2023083

**Published:** 2023-09-03

**Authors:** Pardis Mohammadzadeh, Farhad Moradpour, Bijan Nouri, Farideh Mostafavi, Farid Najafi, Ghobad Moradi

**Affiliations:** 1 Department of Epidemiology and Biostatistics, School of Medicine, Kurdistan University of Medical Sciences, Sanandaj, Iran; 2 Student Research Committee, Kurdistan University of Medical Sciences, Sanandaj, Iran; 3 Social Determinants of Health Research Center, Kurdistan University of Medical Sciences, Sanandaj, Iran; 4 Health Metrics and Evaluation Research Center, Research Institute for Health Development, Kurdistan University of Medical Sciences, Sanandaj, Iran; 5 Department of Epidemiology, School of Public Health and Safety, Shahid Beheshti University of Medical Sciences, Tehran, Iran; 6 Department of Epidemiology, School of Health, Kermanshah University of Medical Sciences, Kermanshah, Iran

**Keywords:** Metabolic syndrome, Social class, Health inequalities, Concentration index, Iran

## Abstract

**OBJECTIVES:**

The worldwide incidence of metabolic syndrome (MetS) has increased in recent decades. In this study, we investigated the socioeconomic inequalities associated with MetS and its components in a sample of the Iranian Kurdish population.

**METHODS:**

We used data from 3,996 participants, aged 35 years to 70 years, from the baseline phase of the Dehgolan Prospective Cohort Study (February 2018 to March 2019). The concentration index and concentration curve were used to measure inequality and the Blinder-Oaxaca decomposition method was used to examine the contribution of various determinants to the observed socioeconomic inequality in MetS and its components.

**RESULTS:**

The prevalence of MetS was 34.44% (95% confidence interval [CI], 32.97 to 35.93). The prevalence of MetS was 26.18% for those in the highest socioeconomic status (SES), compared with 40.51% for participants in the lowest SES. There was a significant negative concentration index for MetS (C=-0.13; 95% CI, -0.16 to -0.09), indicating a concentration of MetS among participants with a lower SES. The most prevalent component was abdominal obesity (59.14%) with a significant negative concentration index (C=-0.21; 95% CI, -0.25 to -0.18). According to decomposition analysis, age, gender, and education were the highest contributing factors to inequality in MetS and its components.

**CONCLUSIONS:**

This study showed socioeconomic inequality in MetS. People with a low SES were more likely to have MetS. Therefore, policymakers and health managers need to develop appropriate strategies to reduce these inequalities in MetS across age groups, genders, and education levels, especially among women and the elderly.

## GRAPHICAL ABSTRACT

[Fig f3-epih-45-e2023083]

## INTRODUCTION

Metabolic syndrome (MetS) is characterized by the coexistence of several cardiovascular risk factors including impaired fasting glucose (IFG), obesity, dyslipidemia, and hypertension (HTN) [1], which are associated with several diseases and premature mortality [2]. The worldwide incidence of MetS has increased in recent decades [2]. Depending on the definition, the prevalence of MetS varies from 12.5% to 31.4% worldwide [3]. According to different definitions of MetS based on gender and region, the prevalence of MetS in Iran ranges from 24.3% to 53.3% [4,5].

The global Sustainable Development Goals (SDG) prioritize the measurement and monitoring of non-communicable disease (NCD) indicators to track progress towards reducing the burden of NCDs and promoting global health. The goal of SDG 3 is a onethird reduction in premature mortality from NCDs by 2030, emphasizing the need to prioritize the prevention and management of NCDs as a key global health and development priority [4,6]. The presence of MetS is a reliable surrogate marker for identifying individuals at high risk for NCDs, as it takes into account the multiple risk factors known to contribute to the development of NCDs and obviates the need to assess each NCD risk factor separately [7].

In addition to reporting, monitoring, and estimating key indicators of the SDG, the importance of assessing inequalities and distribution among subgroups, particularly socioeconomic subgroups, has been emphasized in recent studies on the relationship of inequality to health indicators [8]. Therefore, as a major factor related to the occurrence of NCDs, it is crucial to assess socioeconomic status (SES). To reduce the burden of NCDs, further studies are needed to examine the prevalence and effects of SES on NCDs and to develop comprehensive care programs and effective interventions aimed at preventing their occurrence [9].

In the Third National Health and Nutrition Examination Survey (NHANES III), lower SES was associated with a higher risk of MetS among White, Black, and Mexican American women [10]. In contrast, a study of the population in Spain showed that a lower SES affected the prevalence of MetS in both genders, though women were more frequently affected than men [11].

MetS is a complex disorder influenced by factors such as lifestyle choices, genetics, and SES. Although most studies suggest a reverse association between SES and MetS, other studies have failed to show such an association. To better understand the factors contributing to the development of MetS and its associated chronic diseases, further studies with larger sample sizes are needed [10-14].

Furthermore, the contribution of various independent explanatory variables, including demographic variables, to the observed differences among SES groups is poorly understood [14].

SES, as a key determinant, strongly influences the development of chronic diseases such as MetS [15]. Several studies have shown that individuals with a lower SES are more likely to develop MetS than those with a higher SES due to limited access to healthcare, education, employment, and healthy food options [10,15]. This can also result in the delayed diagnosis and treatment of MetS risk factors, such as HTN and diabetes, thus increasing the risk of developing MetS. The relationship between SES and MetS is bidirectional, with MetS also contributing to a lower SES through decreased productivity, increased healthcare costs, and reduced quality of life [10,12,15,16].

The decomposition of observed inequality can provide health professionals and policymakers with detailed information to target the modifiable risk factors of MetS and better plan health promotion programs [17]. The prevalence of MetS and its components has increased significantly over the past 20 years, with observed shifts in different SES groups [18,19]. As a social phenomenon with a high prevalence and association with chronic diseases, MetS varies across different SES groups in both developing and developed countries [18-21]. Therefore, understanding the social determinants, particularly SES, is crucial for developing effective prevention and management strategies that address the underlying factors contributing to MetS, thus achieving the SDG [4,6].

A study by Liu et al. [13] found that the highest prevalence of MetS has shifted from the higher SES groups in the 2000s to lower SES groups in recent years. Addressing MetS is crucial for reducing the burden of NCDs, promoting global health, and achieving the SDG. Addressing the social determinants of MetS can help reduce health inequalities and promote greater equity. Focused studies are needed to learn more about the distribution of MetS in different communities and subgroups from an economic and social perspective [14].

This study measured socioeconomic inequalities in MetS among Iranian Kurdish adults and identified factors contributing to these inequalities. Using structured SES indicators based on assets, the study decomposed observed inequality and examined the contribution of various explanatory variables. To the best of our knowledge, this is the first study to investigate such inequalities in MetS utilizing the Blinder-Oaxaca (B-O) decomposition method. Our findings provide valuable insight into the socioeconomic determinants of MetS in this population and serve as a baseline for future comparisons across different populations.

## MATERIALS AND METHODS

### Study population

In this cross-sectional study, we used data from the Dehgolan Prospective Cohort Study (DehPCS), a branch of the Prospective Epidemiological Research Studies in Iran (PERSIAN) Cohort Study in Kurdistan Province in the west of Iran [22]. The city of Dehgolan is in the south of Kurdistan Province and has a population of approximately 26,000, almost all of whom are Kurds. The objective of the DehPCS was to identify risk factors for the most prevalent NCDs in the region. The DehPCS enrolled 3,996 participants aged 35 years to 70 years from February 2018 to March 2019 [22]. We excluded 41 individuals from the study (1%), including those with missing data and pregnant women whose complete data on the components of MetS (such as waist circumference) were not available. Detailed information on the DehPCS can be found in the study protocol [22].

### Measurements

We defined MetS according to the National Cholesterol Education Program Adult Treatment Panel III (NCEP ATP-III) criteria [1,23,24]. According to the NCEP ATP-III, MetS is defined as the presence of at least 3 of the following 5 criteria: abdominal obesity (waist circumference ≥ 102 cm for men and ≥ 88 cm for women), IFG (fasting blood glucose ≥ 100 mg/dL or related pharmacological treatment), dyslipidemia based on triglyceride (TG) levels (TG ≥ 150 mg/dL or related pharmacological treatment), dyslipidemia based on high-density lipoprotein (HDL) cholesterol levels (HDL cholesterol < 40 mg/dL for men, < 50 mg/dL for women, or related pharmacologic treatment), and HTN (systolic blood pressure ≥ 130 mmHg and/or diastolic blood pressure ≥ 85 mmHg, or related pharmacologic treatment) [23,24]. Blood pressure was measured in the right arm of each participant in a sitting position (2 separate measurements for right arm systolic and diastolic blood pressure) during the DehPCS baseline phase [22,25].

Income is an important component of SES, but direct wealth and self-reported income can have limitations when calculating SES in developing countries such as Iran. Unfortunately, the data we used for our study from the DehPCS did not include information on income levels. Therefore, we were unable to investigate this factor in our analysis. Instead, we used asset ownership data (e.g., whether the participant owned a car, a motorcycle, and household appliances such as a refrigerator, vacuum cleaner, computer, and washing machine) as well as additional housing characteristics (e.g., a bathroom and separate kitchen in the home) of the participants to create a comprehensive SES variable. Principal component analysis (PCA), as described by Filmer & Pritchett [26], was used to define the asset index [27]. Finally, we divided the asset indices into 5 socioeconomic quintiles, from the lowest (first quintile) to the highest (fifth quintile), according to the comprehensive SES variable created by PCA [27]. The socio-demographic variables included age, gender, education, occupation, and marital status. Pharmacologic treatments were self-reported in response to a question about the use of different types of medications in the first phase of the DehPCS data collection [22].

### Statistical analysis

First, the prevalence of MetS and its components in the population was calculated. Then, socioeconomic inequality was measured using the concentration curve and concentration index. The normalized Wagstaff formula was used to calculate the concentration index as follows:


(1) $$C=\frac{2}{n\mu }\sum_{i=1}^{n}y_{i}R_{i}-1$$

where n is the sample size and R denotes the fractional rank of the person in the asset index from poorest to richest. The symbol mu (*µ*) represents the mean of a binary variable *y_i_* [28].

Briefly, concentration index indicates the extent to which outcomes (MetS in this study) differ among individuals in the SES ranking. The concentration curve is an x-y axis graph with the x-axis showing the cumulative percentage of the sample, ordered by lowest (poorest) SES to highest. On the y-axis, the cumulative percentage of the outcome variable (e.g., MetS) is compared with each cumulative percentage in the SES distribution [27,29]. In general, the farther the curve is from the diagonal of the graph (line of equality), the more pronounced the degree of health inequality [30]. The concentration index takes negative values when the curve is above the diagonal line, which indicates the concentration of outcomes (here MetS) in the groups with low SES. In contrast, the concentration curve below the equality line corresponds to the positive values of the concentration index and indicates the concentration of the outcome in the high SES groups [27,30].

Finally, the B-O decomposition was used to determine the contributions of the various explanatory variables to the observed difference in health variables between the poorest and richest groups. The B-O decomposition indicates the magnitude of the observed difference in the predicted outcome between 2 groups (lowest and highest SES). As mentioned earlier, the original B-O decomposition of the 2-group difference in the average value of the response variable y can be expressed as follows:


(2) $$\begin{array}{cccc} ∆\bar{Y}=\left(\beta_{0}^{1}-\beta_{0}^{2}\right) & +\sum_{j=1}^{k}\beta_{j}^{2}\left({\bar{x}}_{j}^{1}-{\bar{x}}_{j}^{2}\right) & +\sum_{j=1}^{k}{\bar{x}}_{j}^{2}\left(\beta_{j}^{1}-\beta_{j}^{2}\right) & +\sum_{j=1}^{k}\left({\bar{x}}_{j}^{1}-{\bar{x}}_{j}^{2}\right)\left(\beta_{j}^{1}-\beta_{j}^{2}\right)\\ B & E & C & I \end{array}$$

This equation explains the decomposition from the perspective of group 2, with group 1 as the reference. Therefore, it is possible to estimate the contribution of 4 components to the predicted difference: (1) the first component (B) is attributed to fundamental differences; (2) the second component (E) represents differences in the means of the determinants or xj (called the “endowment effect” or “explained”) and represents the portion of the difference that can be explained by differences in the characteristics or attributes of the 2 groups; (3) the third component (C) represents differences in the mean of the coefficients or *β_j_* (called the “coefficient effect” or “unexplained”) and represents the portion of the difference that cannot be explained by differences in how the characteristics affect outcomes; (4) the fourth component (I) represents the interaction differences between xj and *β_j_*, which are any additional effects that arise from interactions between endowments and the coefficient [17,31]. To perform decomposition, we used ordinary logistic regression to examine the association between MetS and its components as outcome variables and explanatory factors such as age, gender, marital status, education, occupation, and SES groups. We included significant factors in the B-O model. In this study, a p< 0.05 was considered to indicate statistical significance [17].

The “conindex” and “oaxaca” commands were used for the concentration index and Oaxaca decomposition analysis in Stata version 17 (StataCorp., College Station, TX, USA) and with the Oaxaca package for the Oaxaca plot in R Studio (Posit, Boston, MA, USA) [17,32] (Supplementary Material 1).

### Ethics statement

The design and implementation of the study were approved by the Ethics Committee of Kurdistan University of Medical Sciences (approval code IR.MUK.REC.1401.072).

## RESULTS

This study included 3,955 participants from the DehPCS. Most participants were women (n= 2,209, 55.8%) and 12.9% had an academic education. The mean age of participants was 48.40±8.92 years. The prevalence of MetS and its components is presented in Table 1 according to the participants’ general characteristics. The prevalence of MetS in the study population was 34.44% (95% CI, 32.97 to 35.93). We found a direct relationship between the prevalence of MetS and age and found a higher prevalence in women. We discovered an inverse relationship between the prevalence of MetS and education level and SES quintile; individuals with a lower education level or a lower SES had a higher prevalence of MetS. The most prevalent components of MetS were abdominal obesity and low HDL (59.14% and 49.75%, respectively). Although MetS, HTN, IFG, and abdominal obesity increase with age, elevated TG levels and low HDL appear to be relatively unchanged across age groups (Table 1).

**Figure 1.**
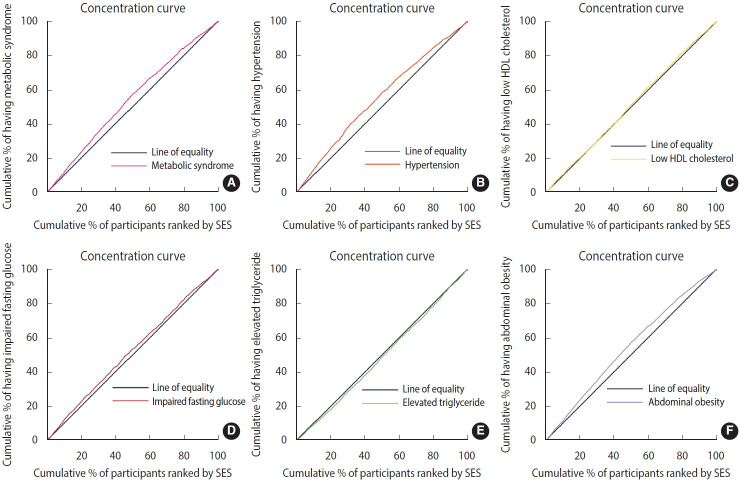
Concentration curves for metabolic syndrome (A) and its components (B) hypertension, (C) Low high-density lipoprotein (HDL) cholesterol, (D) impaired fasting glucose, (E) elevated triglyceride, and (F) abdominal obesity in Dehgolan Prospective Cohort Study.

A significant negative concentration index was found for MetS (C= -0.13; 95% CI, -0.16 to 0.09; p< 0.001) (Table 2). The concentration curve was also above the diagonal line, indicating a higher concentration of MetS in participants with low SES (Figure 1A). We also found a significant negative concentration index for 3 of the 5 components of MetS, including abdominal obesity (C= -0.21; 95% CI, -0.25 to -0.18; p< 0.001), HTN (C= -0.14; 95% CI, -0.18 to -0.11; p<0.001), IFG (C=-0.07; 95% CI, -0.10 to -0.02; p=0.007), and an insignificant negative concentration index for low HDL (C= -0.03; 95% CI, -0.07 to 0.01; p= 0.093). In contrast, a positive concentration index was found for elevated TG levels, indicating a higher concentration in participants with high SES (C= 0.04; 95% CI, 0.01 to 0.08; p= 0.020; Table 2). Figure 1B shows concentration curves for the components of MetS in the study population. The curves above the diagonal line indicate an inequality in favor of the highest SES population for abdominal obesity, HTN, IFG, and low HDL, whereas the curve below the line for elevated TG indicates a pro-poor inequality.

The logistic results for MetS and its components are shown in Table 3. After adjustment for other factors, the odds of MetS increased with age and were 2.67 times higher in women than in men. MetS had an inverse association with education and SES. HTN was directly related to age and some levels of education. Low HDL was related to age, gender, and SES. IFG was associated with age and gender. An elevated TG level was associated with gender, occupation, and SES, and abdominal obesity was associated with SES, gender, education level, and some age groups.

**Figure 2.**
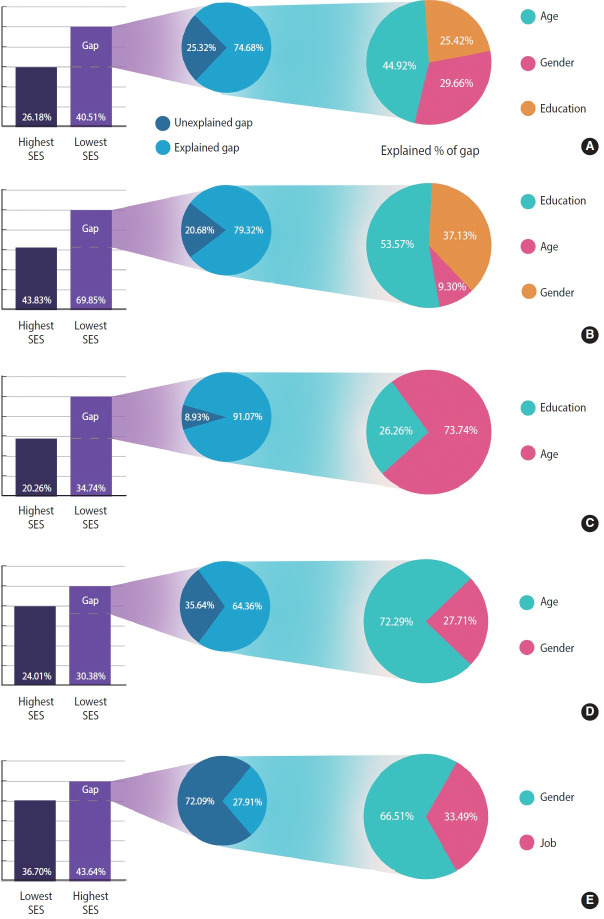
Decomposing the gap in prevalence of (A) metabolic syndrome and its components, (B) abdominal obesity, (C) hypertension, (D) impaired fasting glucose, and (E) elevated triglyceride between highest and lowest socioeconomic status (SES) participants in Dehgolan Prospective Cohort Study.

After measuring inequality, we used B-O decomposition to calculate the contribution of each determinant to the observed inequality in MetS and its components. The difference between the prevalence of MetS in the lowest SES group (40.51%) and in the highest SES group (26.18%) was 14.33% (Table 4). In general, 74.61% of this inequality was due to the explained factors, and 23.67% remained unexplained (Figure 2A, Supplementary Material 2). The most important factors were age (45.13%), gender (29.72%), and education level (25.19%) (Figure 2A). In other words, increased education levels led to a decrease in the observed inequality in MetS by 25.19% of the total study population. Older age, women gender, and a lower education level increased the gap between the 2 economic groups. Figure 2 shows that, among the determinants of inequality in MetS and its components, age, gender, and education level were the greatest contributing factors. For example, the “unexplained part” (13.75%; -0.011/-0.080) of the portion of education in the MetS reflects a difference between 2 groups that cannot be attributed to differences in the covariates or differences in the way that the covariates affect the outcome variable (Table 4). The “interaction part,” which was insignificant in the level of variables in our analysis, refers to the gap that is explained by the interaction between the endowment and coefficient effects.

## DISCUSSION

Our study quantified the socioeconomic inequalities in MetS and its components as well as the contributing factors. We found that 34.44% of the study participants had MetS, and that the prevalence of MetS was higher in women. In general, our study also highlighted the higher prevalence of MetS in individuals with a low SES. The concentration index for MetS was -0.13, which indicated that MetS was more concentrated in the population with a lower SES. Most components of MetS showed similar patterns, except for elevated TG levels. The concentration index of the components including abdominal obesity, HTN, IFG, elevated TG level, and low HDL were -0.21, -0.14, -0.06, 0.04, -0.03, respectively.

According to the B-O decomposition results, 76.33% of the gap between the richest and the poorest groups was caused by socioeconomic determinants or explained factors. Of these factors, age was found to be the most significant contributing factor in MetS inequality, which is not surprising given that MetS is more prevalent in older age groups. Gender was also identified as an important factor, which could be due to the differences in health behaviors and access to healthcare between men and women. Education was another key factor contributing to the observed inequality in MetS, likely because education is a strong predictor of health literacy and health-promoting behaviors, such as healthy eating and physical activity [33-35].

The overall prevalence of MetS in our study was consistent with a meta-analysis of different provinces in Iran conducted by Kalan Farmanfarma et al. [4]. However, our findings differed from the studies by Shafiee et al. [36] and Soofi et al. [37], which used different diagnostic criteria for MetS. This is exemplified by the higher cut-off point for waist circumference in the NCEP_ATP III compared to the International Diabetes Federation (IDF); therefore, the reported prevalence of MetS based on the IDF would be higher in the same population [2]. In addition, as influencing variables, the varied lifestyles in different regions of Iran cannot be disregarded [38].

Our results suggest that MetS is concentrated in populations with a lower SES. This is consistent with the study by Hajian-Tilaki et al. [38] on the Iranian population and the study by Abbate et al. [11] in Spain, both of which showed pro-rich inequality in MetS. In contrast, Mirhosseini et al. [39] and Soofi et al. [37] found opposite results and reported a greater concentration of MetS in the higher SES population. In contrast to our results, Delavar et al. [40] also found a higher prevalence of MetS in individuals from families with a high SES. This heterogeneity in the relationship between SES and MetS could be due to differences in socio-cultural context, lifestyle behaviors, environmental factors, or healthcare access. For example, in some regions, individuals with a lower SES may have limited access to healthcare services, which can result in delayed diagnosis and treatment of MetS risk factors. In other regions, individuals with a lower SES may have limited access to healthy food options, which can increase the risk of developing MetS [41].

Therefore, it is important to consider the socio-cultural context and unique factors that may influence the relationship between SES and MetS in different regions or populations. This highlights the need for tailored interventions and policies that address the social determinants of health and improve access to healthcare, education, and healthy food options and consider the regional and population-specific factors that contribute to the observed socioeconomic inequality in MetS. Ultimately, a better understanding of these complex relationships can help to develop more effective strategies for preventing and managing MetS in different populations [13,15,18].

The B-O decomposition results showed that most differences in the prevalence of MetS can be described by age, gender, and education. Among these variables, education can be considered a modifiable factor, highlighting the importance of education in improving the health of the population in general. Furthermore, for intervention programs to be effective, our decomposition results suggest that the interventions should be targeted toward women and older populations.

Najafi et al. [16] found that not only did the prevalence of MetS vary across Iranian regions, but the concentration index also varied across regions. For example, they found that in Chaharmahal and Bakhtiari Provinces, obesity was concentrated in the lower SES groups, whereas in Khouzestan, it was concentrated in the higher SES groups [16]. They cited differences in education level, physical activity, dietary habits, and sedentary lifestyle as possible explanations for their findings. This finding supports our results and highlights the importance of conducting region-specific studies to determine the etiology of MetS and its components. Demographic differences among regions can be considered a source of the differences in region-specific outcomes. Factors such as environmental influences [42], climate, culture, and social relations can also have an impact and should not be neglected [43].

We found a higher prevalence of abdominal obesity in participants with a lower SES and that, as determining factors, education level, gender, and age contributed most to the gap between the lowest and highest SES. This finding is consistent with a review of Iranian obesity studies, in which older age, lower education level, and women gender were associated with a higher risk of obesity [36]. However, a study of the Iranian population by Najafi et al. [16] found that adults who were overweight and obese were mainly of high SES, which is in contrast to our findings. People with low SES tend not to have the benefit of a healthy diet and tend to consume more carbohydrates and unhealthy foods [44], which can lead to problems with being overweight or obese. Lower health literacy in low socioeconomic groups also limits their knowledge and understanding of preventive measures and approaches to weight control [16,36]. In addition, some of the differences between our results and those of other studies of the Iranian population could be due to the use of differing obesity measures. Apart from the role of physical inactivity, the role of a high-carbohydrate diet (which is usually cheaper than a high-protein diet) in individuals with a low SES has been highlighted as a factor in the higher prevalence of MetS and its components [33].

In our study, HTN contributed to the second-highest level of inequality among the components of MetS. Although similar results were found in the Spanish study and in the Whitehall II study [11,45], different results were demonstrated in the study by Shafiee et al. [36]. Studies have shown that individuals with a lower SES are more likely to have HTN and to experience worse outcomes from the condition. This is due to a variety of factors, including limited access to healthcare, unhealthy diets, and higher levels of stress. In addition, those with lower incomes may not be able to afford medications or treatments for HTN, making it even more difficult for them to manage the condition [9,46,47].

Our investigation revealed a pro-rich inequality for IFG, which is consistent with the study by Peykari et al. [48], but contrasts with the research by Shafiee et al. [36]. We found that individuals with a lower SES were more likely to have IFG than those with a higher SES. The best explanations may include limited access to healthcare and lifestyle factors such as unhealthy diets and sedentary behavior among individuals with a lower SES. Furthermore, individuals with a lower SES may have a higher prevalence of the comorbidities associated with IFG, such as obesity and HTN (again, due to limited access to healthy foods and resources for physical activity) [33,35].

Therefore, addressing the social determinants of health, such as improving access to healthcare, healthy food options, and resources for physical activity, could be effective in reducing the burden of IFG, particularly in individuals with a lower SES. Our findings underscore the need for tailored interventions and policies that consider the unique factors that contribute to the socioeconomic inequality in IFG. Ultimately, a better understanding of these complex relationships can help in developing more effective strategies for the prevention and management of IFG in different populations, especially those with a lower SES [46].

Our research revealed that individuals with a higher SES tend to have higher levels of TG. This result is consistent with the study by Shafiee et al. [36]. People with a higher SES may be more sedentary and less physically active, both of which contribute to elevated TG levels. Therefore, it is important for people with a high SES to maintain a healthy diet and lifestyle to reduce their risk of developing these conditions [49].

Addressing MetS and reducing the NCD burden are key priorities for global health and development, as highlighted by the SDG. Measuring and monitoring the prevalence of MetS can serve as an effective proxy measure for NCDs and help identify individuals at high risk for developing these diseases. However, it is equally important to assess disparities in MetS and NCDs across different socioeconomic groups and areas, as socioeconomic inequalities persist in health outcomes. By continuously measuring and monitoring these disparities, we can identify and address the underlying social determinants of health and develop more effective interventions to promote health equity for all [6,13,50].

In addition to the strengths of our study, including its sample size and inclusion of the components of MetS, our study also had some limitations. First, the cross-sectional design of the study makes it difficult to determine a causal association between MetS and the independent variables. Secondly, our study did not include some important determinants, such as cultural and regional factors. Those who did not have 3 or more components of MetS were excluded from our study. Although this likely did not affect our results, it should be considered when interpreting the results.

In conclusion, our study found that the prevalence of MetS in the study area was relatively high compared to other studies in Iran, and that MetS occurs mainly in individuals with a lower SES. Our study suggests that the observed inequality is mainly due to age, gender, and education, and that future efforts to reduce the risk factors of MetS should target those affected by socioeconomic inequality. Education is needed for those with a lower SES, especially for women and the elderly. Increased awareness is needed among people with a higher SES as they age. Both are necessary to eliminate the socioeconomic gaps in the development of MetS and to ensure that all groups benefit.

## DATA ACCESSIBILITY

The data from this study are not freely accessible, but to increase scientific output, the study data can be accessed for secondary analysis by submitting a request under the terms and conditions for publication of PERSIAN cohort data. Information on how to access the data (e.g., authorship rules, data transfer agreements and samples, and the data dictionary) is available on the PERSIAN cohort website at https://persiancohort.com.

## Figures and Tables

**Figure f3-epih-45-e2023083:**
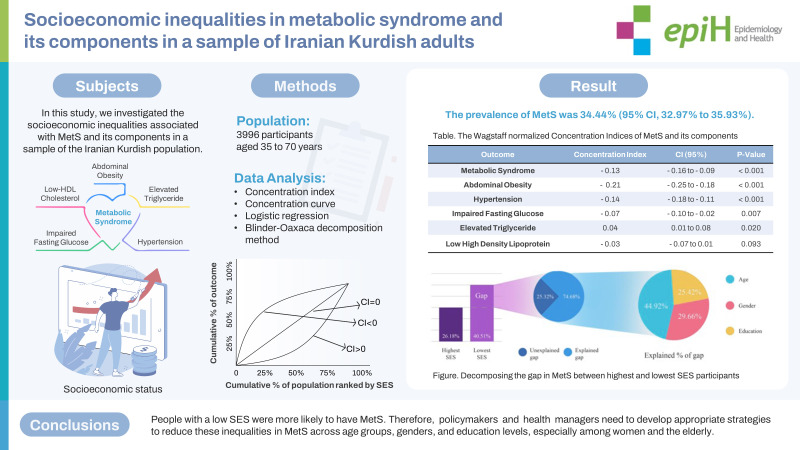


**Table 1.** Distribution of metabolic syndrome (MetS) and its components according to the demographic and socioeconomic factors of participants in the Dehgolan Prospective Cohort Study

Varialbels		n (%)	MetS	HTN	IFG	Elevated TG	Low HDL	Abdominal obesity
All		3,955 (100)	34.44 (32.97, 35.93)	26.83 (25.47, 28.24)	26.85 (25.48, 28.28)	40.70 (39.15, 42.26)	49.75 (48.17, 51.33)	59.14 (57.60, 60.67)
Age (yr)								
	35-45	1,751 (44.3)	25.81 (23.82, 27.92)	12.43 (10.96, 14.06)	18.29 (16.52, 20.19)	38.49 (36.21, 40.82)	53.22 (50.85, 55.58)	55.33 (52.99, 57.65)
	46-55	1,311 (33.1)	36.69 (34.12, 39.34)	29.02 (26.62, 31.54)	29.19 (26.76, 31.74)	42.82 (40.13, 45.56)	47.61 (44.88, 50.35)	61.85 (59.18, 64.45)
	56-65	713 (18.0)	46.14 (42.51, 49.82)	48.45 (44.78, 52.13)	38.82 (35.25, 42.50)	42.01 (38.39, 45.72)	45.10 (41.43, 48.82)	62.03 (58.40, 65.52)
	66-70	180 (4.5)	55.56 (48.23, 62.65)	65.00 (57.75, 71.61)	45.76 (38.56, 53.14)	41.48 (34.43, 48.89)	50.00 (42.66, 57.34)	65.00 (57.75, 71.61)
Gender								
	Men	1,746 (44.1)	21.25 (19.39, 23.23)	24.10 (22.15, 26.18)	23.50 (21.55, 25.58)	43.78 (41.44, 46.15)	35.43 (33.19, 37.73)	25.50 (23.51, 27.60)
	Women	2,209 (55.8)	44.86 (42.80, 46.94)	28.97 (27.12, 30.90)	29.51 (27.62, 31.47)	38.25 (36.22, 40.33)	61.10 (59.02, 63.14)	85.74 (84.22, 87.14)
Marital status								
	Single	44 (1.1)	20.45 (11.00, 34.86)	13.64 (6.26, 27.20)	13.64 (6.25, 27.20)	27.27 (16.19, 42.14)	54.55 (39.86, 68.48)	50.00 (35.64, 64.36)
	Married	3,632 (91.8)	33.23 (31.72, 34.78)	25.62 (24.22, 27.07)	26.04 (24.62, 27.51)	40.77 (39.16, 42.40)	49.28 (47.63, 50.93)	57.14 (55.52, 58.74)
	Divorced/widow	279 (7.1)	52.33 (46.46, 58.13)	44.60 (38.86, 50.50)	39.56 (33.93, 45.48)	41.91 (36.19, 47.86)	55.15 (49.19, 60.96)	86.69 (82.17, 90.20)
Education								
	Illiterate	1,238 (31.3)	50.32 (47.54, 53.11)	41.94 (39.22, 44.72)	35.54 (32.89, 38.28)	40.85 (38.10, 43.65)	55.01 (52.19, 57.80)	83.31 (81.12, 85.29)
	Middle school	1,602 (40.5)	31.46 (29.23, 33.78)	21.96 (19.99, 24.06)	24.53 (22.46, 26.73)	40.20 (37.80, 42.66)	49.58 (47.11, 52.06)	55.84 (53.39, 58.26)
	High school	604 (15.3)	23.34 (20.14, 26.89)	16.19 (13.45, 19.37)	20.65 (17.56, 24.12)	39.18 (35.31, 43.20)	47.61 (43.59, 51.66)	45.42 (41.48, 49.43)
	Academic level	511 (12.9)	18.40 (15.27, 22.00)	17.86 (14.75, 21.45)	20.32 (17.01, 24.09)	43.66 (39.36, 48.06)	40.04 (35.82, 44.42)	27.02 (23.33, 31.06)
Job								
	No	1,944 (49.5)	45.78 (43.58, 48.00)	31.94 (29.90, 34.05)	30.59 (28.55, 32.70)	39.98 (37.79, 42.21)	59.55 (57.31, 61.74)	82.77 (81.02, 84.38)
	Yes	1,986 (50.5)	23.51 (21.70, 25.43)	21.86 (20.09, 23.74)	23.23 (21.40, 25.17)	41.49 (39.31, 43.70)	40.34 (38.18, 42.54)	36.16 (34.07, 38.30)
SES								
	Lowest	780 (19.9)	40.51 (37.12, 44.00)	34.74 (31.48, 38.16)	30.38 (27.21, 33.75)	36.82 (33.45, 40.32)	50.33 (46.77, 53.89)	69.85 (66.52, 72.97)
	Low	783 (20.0)	38.19 (34.85, 41.64)	29.31 (26.21, 32.60)	27.69 (24.63, 30.98)	40.74 (37.30, 44.27)	49.67 (46.13, 53.22)	66.84 (63.46, 70.05)
	Middle	782 (20.0)	36.57 (33.27, 40.01)	26.83 (23.83, 30.05)	26.08 (23.08, 29.31)	42.39 (38.92, 45.93)	53.54 (49.99, 57.06)	60.56 (57.09, 63.94)
	High	784 (20.0)	31.25 (28.10, 34.58)	22.76 (19.96, 25.83)	26.56 (23.56, 29.80)	40.26 (36.85, 43.77)	50.46 (46.92, 53.98)	55.23 (51.73, 58.68)
	Highest	783 (20.0)	26.18 (23.22, 29.38)	20.26 (17.58, 23.22)	24.01 (21.11, 27.18)	43.64 (40.16, 47.19)	45.14 (41.64, 48.70)	43.83 (40.38, 47.34)

Values are presented as prevalence % (95% confidence interval). HTN, hypertension; HDL, high-density lipoprotein; IFG, impaired fasting glucose; TG, triglyceride; SES, socioeconomic status.

**Table 2.** The Wagstaff normalized concentration indices of metabolic syndrome and its components among Dehgolan Prospective Cohort Study participants

Outcomes	Concentration index (95% CI)	p-value
Metabolic syndrome	-0.13 (-0.16, -0.09)	<0.001
Components of metabolic syndrome		
Abdominal obesity	-0.21 (-0.25, -0.18)	<0.001
Hypertension	-0.14 (-0.18, -0.11)	<0.001
Impaired fasting glucose	-0.07 (-0.10, -0.02)	0.007
Elevated triglyceride level	0.04 (0.01, 0.08)	0.020
Low high-density lipoprotein	-0.03 (-0.07, 0.01)	0.093

**Table 3.** The association between socioeconomic variables and MetS and its components in logistic regression among Dehgolan Prospective Cohort Study participants^1^

Variables		Crude	Adjusted	Adjusted				
		MetS		HTN	Low HDL	IFG	Elevated TG	Abdominal obesity
Age (yr)								
	35-45	1.00 (reference)	1.00 (reference)	1.00 (reference)	1.00 (reference)	1.00 (reference)	1.00 (reference)	1.00 (reference)
	46-55	1.67 (1.43, 1.94)^*^	1.60 (1.34, 1.92)^*^	2.67 (2.17, 3.27)^*^	0.82 (0.70, 0.97)^*^	1.78 (1.47, 2.15)^*^	1.12 (0.96, 1.32)	1.49 (1.21, 1.83)^*^
	56-65	2.46 (2.05, 2.95)^*^	2.30 (1.84, 2.89)^*^	5.65 (4.43, 7.21)^*^	0.74 (0.59, 0.92)^*^	2.78 (2.19, 3.51)^*^	1.07 (0.87, 1.33)	1.18 (0.90, 1.56)
	66-70	3.59 (2.63, 4.91)^*^	3.41 (2.35, 4.95)^*^	10.78 (7.31, 15.90)^*^	1.00 (0.70, 1.44)	3.61 (2.49, 5.23)^*^	0.99 (0.69, 1.42)	1.45 (0.92, 2.28)
Gender								
	Men	1.00 (reference)	1.00 (reference)	1.00 (reference)	1.00 (reference)	1.00 (reference)	1.00 (reference)	1.00 (reference)
	Women	3.02 (2.62, 3.48)^*^	2.67 (2.13, 3.36)^*^	1.16 (0.90, 1.50)	2.80 (2.26, 3.48)^*^	1.40 (1.10, 1.78)^*^	0.68 (0.55, 0.84)^*^	14.29 (11.04, 18.50)^*^
Marital status								
	Single	0.57 (0.27, 1.21)	0.91 (0.37, 2.22)	0.78 (0.42, 1.44)	0.60 (0.25, 1.45)	0.68 (0.34, 1.34)	0.34 (0.18, 0.66)^*^	0.57 (0.27, 1.21)
	Married	1.00 (reference)	1.00 (reference)	1.00 (reference)	1.00 (reference)	1.00 (reference)	1.00 (reference)	1.00 (reference)
	Divorced/widow	1.02 (0.78, 1.35)	1.05 (0.78, 1.40)	0.87 (0.66, 1.15)	1.16 (0.88, 1.55)	1.19 (0.91, 1.57)	1.10 (0.72, 1.66)	1.02 (0.78, 1.35)
Education								
	Illiterate	4.49 (3.49, 5.77)^*^	1.77 (1.24, 2.51)^*^	1.27 (0.87, 1.86)	1.15 (0.84, 1.58)	1.23 (0.85, 1.77)	1.08 (0.79, 1.47)	4.22 (2.87, 6.20)^*^
	Middle school	2.04 (1.59, 2.61)^*^	1.42 (1.05, 1.91)^*^	0.96 (0.70, 1.32)	1.11 (0.84, 1.44)	1.11 (0.82, 1.50)	0.96 (0.75, 1.24)	2.15 (1.58, 2.91)^*^
	High school	1.35 (1.01, 1.81)^*^	1.19 (0.87, 1.64)	0.96 (0.68, 1.35)	1.10 (0.84, 1.44)	1.09 (0.79, 1.50)	0.91 (0.70, 1.18)	1.83 (1.33, 2.51)^*^
	Academic level	1.00 (reference)	1.00 (reference)	1.00 (reference)	1.00 (reference)	1.00 (reference)	1.00 (reference)	1.00 (reference)
Job								
	No	2.75 (2.39, 3.15)^*^	1.15 (0.94, 1.42)	1.14 (0.90, 1.44)	1.08 (0.88, 1.32)	0.99 (0.80, 1.25)	1.23 (1.01, 1.51)^*^	1.12 (0.87, 1.43)
	Yes	1.00 (reference)	1.00 (reference)	1.00 (reference)	1.00 (reference)	1.00 (reference)	1.00 (reference)	1.00 (reference)
SES								
	Lowest	1.92 (1.55, 2.38)^*^	0.71 (0.53, 0.93)^*^	0.92 (0.68, 1.24)	0.79 (0.61, 1.02)	0.76 (0.57, 1.01)	0.72 (0.56, 0.93)^*^	0.56 (0.41, 0.77)^*^
	Low	1.74 (1.41, 2.16)^*^	0.77 (0.59, 1.00)	0.89 (0.66, 1.19)	0.82 (0.64, 1.05)	0.77 (0.59, 1.02)	0.88 (0.69, 1.12)	0.66 (0.48, 0.89)^*^
	Middle	1.63 (1.31, 2.02)^*^	0.89 (0.69, 1.15)	0.92 (0.70, 1.23)	1.08 (0.86, 1.38)	0.79 (0.60, 1.04)	0.95 (0.75, 1.20)	0.75 (0.57, 1.01)
	High	1.28 (1.03, 1.60)^*^	0.88 (0.68, 1.13)	0.90 (0.69, 1.19)	1.06 (0.84, 1.33)	0.94 (0.72, 1.21)	0.88 (0.70, 1.09)	0.87 (0.66, 1.14)
	Highest	1.00 (reference)	1.00 (reference)	1.00 (reference)	1.00 (reference)	1.00 (reference)	1.00 (reference)	1.00 (reference)

Values are presented as odds ratio (95% confidence interval). MetS, metabolic syndrome; HTN, hypertension; HDL, high-density lipoprotein; IFG, impaired fasting glucose; TG, triglyceride; SES, socioeconomic status.

1 All variables (age, gender, marital status, education, job, SES) have been adjusted in the “adjusted odds ratios.”

* p<0.05.

**Table 4.** Results of the Blinder-Oaxaca decomposition of MetS and its components, stratified by highest and lowest SES quintile among Dehgolan Prospective Cohort Study participants

Variables			MetS	HTN	IFG	Elevated TG	Abdominal obesity
Overall							
	Prevalence in 1st SES quintile		0.405 (0.370, 0.440)^*^	0.347 (0.314, 0.381)^*^	0.304 (0.271, 0.336)^*^	0.367 (0.332, 0.401)^*^	0.698 (0.669, 0.728)^*^
	Prevalence in 5th SES quintile		0.262 (0.231, 0.292)^*^	0.202 (0.175, 0.230)^*^	0.240 (0.210, 0.270)^*^	0.436 (0.401, 0.472)^*^	0.438 (0.405, 0.471)^*^
	Difference (total gap)		0.143 (0.097, 0.189)^*^	0.145 (0.101, 0.188)^*^	0.063 (0.019, 0.108)^*^	-0.069 (-0.119, 0.020)^*^	0.260 (0.216, 0.304)^*^
	Endowments (explained)		0.236 (0.139, 0.333)^*^	0.179 (0.085, 0.273)^*^	0.083 (0.021, 0.144)^*^	-0.044 (-0.078, -0.010)^*^	0.387 (0.337, 0.436)^*^
	Coefficients (unexplained)		-0.080 (-0.163, 0.002)	-0.017 (-0.154, 0.119)	-0.046 (-0.095, 0.003)	-0.114 (-0.171, -0.056)^*^	-0.101 (-0.188, -0.014)^*^
	Interaction		-0.012 (-0.135, 0.110)	-0.017 (-0.179, 0.145)	0.026 (-0.042, 0.095)	0.088 (0.041, 0.136)^*^	-0.026 (-0.116, 0.065)
Due to endowments (explained)							
	Age (yr)						
		35-45	0.024 (0.012, 0.037)^*^	0.027 (0.014, 0.041)^*^	0.020 (0.008, 0.032)^*^	N/A	0.011 (0.002, 0.019)^*^
		46-55	0.016 (0.003, 0.030)^*^	0.028 (0.013, 0.043)^*^	0.004 (-0.008, 0.016)	N/A	0.002 (-0.007, 0.012)
		56-65	-0.001 (-0.008, 0.006)	0.003 (-0.004, 0.011)	0.003 (-0.003, 0.009)	N/A	0.001 (-0.004, 0.005)
		66-70	0.067 (0.032, 0.102)^*^	0.074 (0.038, 0.109)^*^	0.033 (-0.002, 0.069)	N/A	0.022 (-0.004, 0.049)
	Gender						
		Men	0.035 (0.019, 0.051)^*^	N/A	0.011 (-0.004, 0.026)	-0.044 (-0.068, -0.021)^*^	0.072 (0.045, 0.098)^*^
		Women	0.035 (0.019, 0.051)^*^	N/A	0.011 (-0.004, 0.026)	-0.044 (-0.068, -0.021)^*^	0.072 (0.045, 0.098)^*^
	Education						
		Illiterate	0.028 (-0.046, 0.103)	0.030 (-0.040, 0.100)	N/A	N/A	0.134 (0.053, 0.216)^*^
		Middle school	0.001 (-0.005, 0.007)	-0.001 (-0.007, 0.006)	N/A	N/A	-0.004 (-0.011, 0.002)
		High school	-0.002 (-0.016, 0.011)	0.003 (-0.012, 0.018)	N/A	N/A	0.009 (-0.005, 0.023)
		Academic level	0.033 (0.000, 0.065)^*^	0.015 (-0.015, 0.044)	N/A	N/A	0.068 (0.047, 0.090)^*^
	Job						
		No	N/A	N/A	N/A	0.022 (-0.003, 0.048)	N/A
		Yes	N/A	N/A	N/A	0.022 (-0.003, 0.048)	N/A
Due to coefficients (unexplained)							
	Age (yr)						
		35-45	0.014 (-0.006, 0.033)	0.001 (-0.017, 0.018)	0.006 (-0.020, 0.031)	N/A	0.011 (-0.009, 0.032)
		46-55	0.001 (-0.025, 0.027)	0.015 (-0.023, 0.053)	-0.016 (-0.053, 0.020)	N/A	-0.020 (-0.051, 0.012)
		56-65	0.004 (-0.007, 0.015)	0.004 (-0.007, 0.014)	-0.003 (-0.018, 0.012)	N/A	0.003 (-0.010, 0.017)
		66-70	-0.002 (-0.004, 0.001)	-0.001 (-0.004, 0.002)	0.001 (-0.002, 0.004)	N/A	-0.001 (-0.003, 0.002)
	Gender						
		Men	-0.019 (-0.043, 0.004)	N/A	-0.017 (-0.044, 0.010)	-0.072 (-0.122, -0.021)^*^	-0.009 (-0.035, 0.017)
		Women	0.010 (-0.002, 0.022)	N/A	0.009 (-0.005, 0.023)	0.037 (0.011, 0.064)^*^	0.005 (-0.009, 0.019)
	Education						
		Illiterate	0.001 (-0.004, 0.004)	0.001 (-0.003, 0.004)	N/A	N/A	-0.006 (-0.014, 0.003)
		Middle school	0.008 (-0.009, 0.025)	0.009 (-0.023, 0.040)	N/A	N/A	0.019 (-0.004, 0.041)
		High school	0.003 (-0.020, 0.027)	-0.012 (-0.038, 0.014)	N/A	N/A	0.014 (-0.013, 0.041)
		Academic level	-0.023 (-0.100, 0.053)	0.002 (-0.070, 0.074)	N/A	N/A	0.010 (-0.074, 0.095)
	Job						
		No	N/A	N/A	N/A	0.001 (-0.019, 0.021)	N/A
		Yes	N/A	N/A	N/A	-0.003 (-0.060, 0.054)	N/A
	_cons		-0.077 (-0.136, -0.017)^*^	-0.036 (-0.168, 0.097)	-0.024 (-0.081, 0.032)	-0.078 (-0.132, -0.023)^*^	-0.128 (-0.212, -0.043)^*^
Due to interaction							
	Age (yr)						
		35-45	0.003 (-0.038, 0.044)	0.009 (-1.339, 1.357)	-0.002 (-0.012, 0.007)	N/A	-0.001 (-0.005, 0.002)
		46-55	0.001 (-0.005, 0.006)	0.124 (-19.277, 19.525)	0.005 (-0.010, 0.021)	N/A	0.002 (-0.003, 0.007)
		56-65	-0.001 (-0.014, 0.013)	-0.040 (-6.373, 6.292)	-0.001 (-0.008, 0.005)	N/A	0.001 (-0.002, 0.002)
		66-70	0.008 (-0.107, 0.123)	0.259 (-40.251, 40.769)	0.006 (-0.024, 0.036)	N/A	-0.001 (-0.010, 0.007)
	Gender						
		Men	-0.005 (-0.083, 0.072)	N/A	0.009 (-0.010, 0.029)	0.043 (0.013, 0.072)^*^	0.001 (-0.004, 0.007)
		Women	-0.005 (-0.083, 0.072)	N/A	0.009 (-0.010, 0.029)	0.043 (0.013, 0.072)^*^	0.001 (-0.004, 0.007)
	Education						
		Illiterate	-0.001 (-0.019, 0.018)	-0.130 (-19.409, 19.149)	N/A	N/A	-0.024 (-0.091, 0.042)
		Middle school	-0.001 (-0.020, 0.017)	-0.073 (-11.398, 11.251)	N/A	N/A	0.002 (-0.001, 0.005)
		High school	0.001 (-0.018, 0.020)	-0.220 (-34.587, 34.147)	N/A	N/A	-0.003 (-0.012, 0.006)
		Academic level	-0.011 (-0.136, 0.115)	0.054 (-9.871, 9.979)	N/A	N/A	-0.003 (-0.030, 0.024)
	Job						
		No	N/A	N/A	N/A	0.001 (-0.029, 0.032)	N/A
		Yes	N/A	N/A	N/A	0.001 (-0.029, 0.032)	N/A
Contributions, %							
	Difference (total gap)		14.33	14.48	6.37	6.94	26.02
		Explained	74.68	91.70	64.36	27.91	79.32
		Unexplained	25.32	8.93	35.64	72.09	20.68
Share of variables due to explained, %							
	Age		44.92	73.74	72.29	-	9.30
	Education		25.42	26.26	-	-	53.57
	Gender		29.66	-	27.71	66.51	37.13
	Job		-	-	-	33.49	-

MetS, metabolic syndrome; SES, socioeconomic status; HTN, hypertension; HDL, high-density lipoprotein; IFG, impaired fasting glucose; TG, triglyceride; N/A, not applicable for variables that had no significant association with outcome variable.

* p<0.05.

## SUPPLEMENTARY MATERIALS

Supplementary Material 1. STATA and R Codes Appendix

Supplementary Material 2 Contributions of differences in Metabolic Syndrome and in coefficients to lowest socioeconomic status (SES), highest SES Oaxaca Decomposition
