# Supplementary material for: Socioeconomic inequalities in metabolic syndrome and its components in a sample of Iranian Kurdish adults
Source: Epidemiol Health. 2023 Sep 3;45:e2023083. doi: 10.4178/epih.e2023083 (PMC10867515; doi:10.4178/epih.e2023083)
Supplement: Supplementary Material 1. — STATA and R Codes Appendix [file epih-45-e2023083-Supplementary-1.docx]

Supplementary Material 1. STATA and R Codes Appendix

| STATA commands: | | |
| --- | --- | --- |
| No. | Command | Description |
| 1 | conindex varname, rank(SES) bounded limits(0 1) wagstaff | This command is used to calculate concentration indices for binary outcome. |
| 2 | clorenz varname, rank(SES) | This command is used to plot concentration curves. |
| 3 | logit depvar i.indepvars | This command is used to estimate logistic regression models. |
| 4 | oaxaca varname normalize(var1?) normalize(var2?) normalize(var3?), by (SES2) logit noisily relax | This command is used to perform the Blinder-Oaxaca Decomposition model for binary outcome. |
| R package and codes: | | |
| No. | Package and Code | Description |
| 1 | oaxaca (formula = depvar ~ var1 + var2 + dummyvar3 + dummyvar4 + dummyvar5 \| SES2 \| dummyvar3 + dummyvar4 + dummyvar5, data = data-file , R = 1000) | This package provides functions for performing the Blinder-Oaxaca Decomposition model. |
| 2 | plot(results, components = c("endowments","coefficients")) | This function is used to plot the results of the Blinder-Oaxaca Decomposition model. |
