# Supplementary material for: Socioeconomic inequalities in metabolic syndrome and its components in a sample of Iranian Kurdish adults
Source: Epidemiol Health. 2023 Sep 3;45:e2023083. doi: 10.4178/epih.e2023083 (PMC10867515; doi:10.4178/epih.e2023083)
Supplement: Supplementary Material 2 — Contributions of differences in Metabolic Syndrome and in coefficients to lowest socioeconomic status (SES), highest SES Oaxaca Decomposition [file epih-45-e2023083-Supplementary-2.docx]

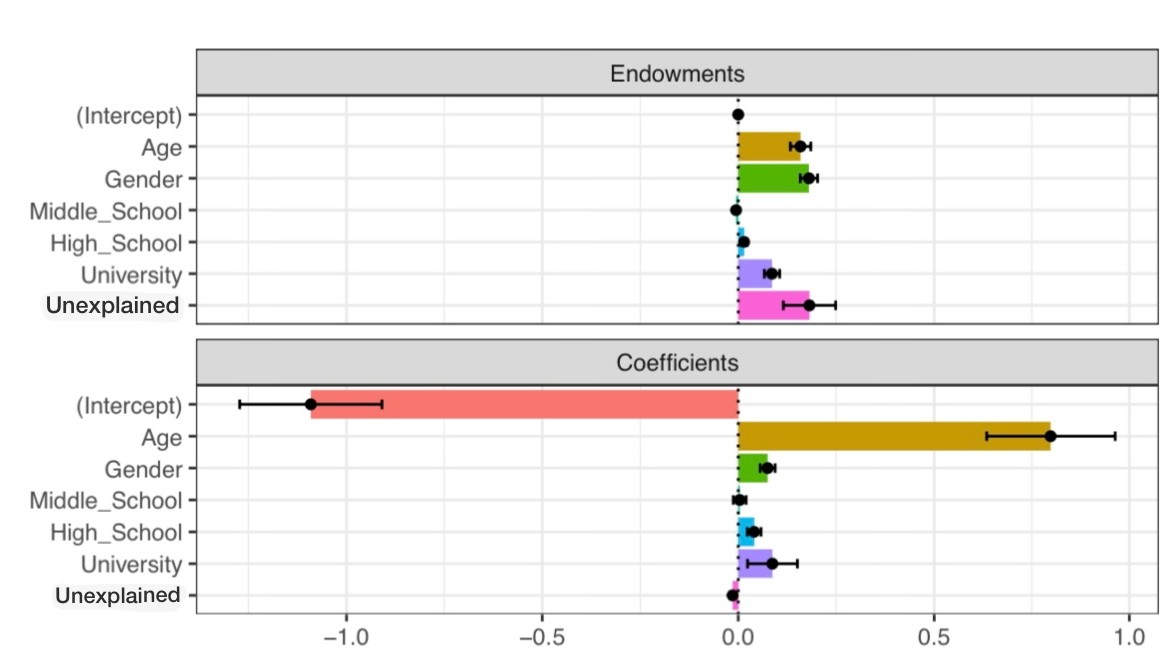


**Supplementary Material 2.** Contributions of differences in Metabolic Syndrome and in coefficients to lowest socioeconomic status (SES), highest SES Oaxaca Decomposition
